# A Case Report on Paget-Schroetter Syndrome Presenting as Acute Localized Rhabdomyolysis

**DOI:** 10.5811/cpcem.2020.6.47335

**Published:** 2020-07-14

**Authors:** Jonathan B. Lee, Ami Kurzweil, Shadi Lahham

**Affiliations:** University of California, Irvine Medical Center, Department of Emergency Medicine, Orange, California

**Keywords:** Paget-Schroetter syndrome, point-of-care ultrasound, axillary vein stenosis

## Abstract

**Introduction:**

The life- or limb-threatening differential diagnosis for upper extremity swelling can include deep vein thrombosis (DVT), infectious processes, and compartment syndrome. Chronic anatomic abnormalities such as axillary vein stenosis are rarely a consideration in the emergency department.

**Case Report:**

We present a 26-year-old female with history of Chiari type 1 malformation who presented with acute left arm swelling. Initial workup, including point-of-care ultrasound, revealed the presence of significant soft tissue swelling without evidence of DVT.

**Conclusion:**

Further workup revealed an early, localized rhabdomyolysis secondary to axillary vein stenosis or venous thoracic outlet syndrome, also known as Paget-Schroetter syndrome.

## INTRODUCTION

Venous stenosis can present with a number of clinical symptoms, including swelling, pain, numbness, discoloration, and paresthesias.^1^ These non-specific symptoms can often lead physicians to consider more common life-threatening conditions such as venous thrombosis, lymphedema, or inflammatory processes. Notably, the upper extremities are more commonly affected by venous stenosis than the lower extremities, and among the most common vein to be affected is the axillary vein.^2^ Venous strictures have been reported to be secondary to fibrosis from placement of central lines, prior radiation, trauma, or extrinsic compression from musculoskeletal structures. One known cause of axillary vein stenosis is venous thoracic outlet syndrome, also known as Paget-Schroetter syndrome.^3^ This condition typically presents in patients whose work or activities require prolonged repetitive motions of the arm, or in a patient with an acute traumatic injury to an upper extremity. Thoracic outlet syndrome has even been reported in patients with cervicothoracic scoliosis and post spinal surgery patients,^4^,^5^ such as seen in this case report.

## CASE REPORT

A 26-year-old female with history of Chiari type 1 malformation, status post intracranial shunt and scoliosis leading to cervical spinal fusion at age 10 presented with left upper arm swelling for seven hours. She stated there was mild, pressure-like sensation and endorsed associated radiating numbness to her fingertips. She denied any pain, but explained she had chronic baseline sensation deficits on the left side of her body as a sequelae from her cervical spinal fusion and thus had diminished ability to sense pain to her left upper extremity since the age of 10. She did not recall any inciting insult, repetitive movement during work or exercise, or trauma to the arm. The patient stated she had an etonogestrel/ethinyl estradiol vaginal ring placed approximately three months earlier and had not had any complications. Her only risk factor for venous thromboembolism was her contraception.

Upon arrival, her vital signs were all within normal limits. On physical exam she was calm and in no acute distress. Her left upper extremity was swollen circumferentially from the distal deltoid to the antecubital fossa, with the greatest area of swelling on the posterior-medial aspect of the left upper extremity overlying the triceps. Her left upper extremity had full passive and active range of motion without pain. Although she had decreased sensation, she reported no change from her baseline complications post cervical spinal fusion. She had 2+ distal radial and ulnar pulses. There was no increased warmth or erythema when compared to the right upper extremity, and she was without ecchymosis. She had five out of five grip strength bilaterally. Her left upper medial posterior compartment was moderately tense. The rest of her extremities had five out of five strength bilaterally.

Based on physical exam, the leading life-threatening diagnosis was upper extremity deep vein thrombosis (DVT). However, the patient had no other risk factors or clinical signs of DVT except for marked upper extremity swelling and etonogestrel/ethinyl estradiol vaginal ring. She was saturating well on room air, without any tachycardia or pleuritic chest pain, and thus initial suspicion for acute pulmonary embolism (PE) was low. Infection was thought to be unlikely given there was no overlying erythema, no warmth, and no systemic signs of infection. Compartment syndrome was considered but thought to be less likely given her compartments were mostly soft, and there was no history of trauma. Although, given the patient’s baseline sensory deficits, occult trauma was still thought to be possible. Lymphedema was seen as less likely without any history of prior surgeries in the axilla that would place her at risk.

Initial work-up consisted of a complete blood count (CBC), basic metabolic panel (BMP), coagulation panel, a formal left upper extremity (LUE) DVT ultrasound performed by an ultrasound technician, and an electrocardiogram (ECG). The ECG was obtained to evaluate for any subtle signs of PE, given a diagnosis of DVT was in consideration. Her CBC and BMP were within normal limits. Her ECG revealed normal sinus rhythm, without evidence of right heart strain. Her formal LUE DVT ultrasound was negative for any venous thrombus (Image, panels 1 and 2).

This initial negative work-up prompted a point-of-care ultrasound (POCUS) to further evaluate the cause of swelling. Images were notable for soft tissue edema including pockets of interstitial fluid between the muscle bellies and perivascular fluid around veins of the upper arm, which upon review were also noticeable in images obtained by the ultrasound technician (Image, panel 3). These findings indicated a possible inflammatory process or a source of venous congestion from a more proximal source. Furthermore, POCUS revealed there were muscle fibers of mixed echogenicity along with notable disorganized muscle fibers, which upon review were also notable in images obtained from the ultrasound technician (Image, panel 4), which could have been consistent with rhabdomyolysis.^6^ These findings prompted a D-dimer, which when resulted as abnormal, prompted both a computed tomography (CT) venography of the chest and upper extremities and CT angiogram of the chest. To assess for potential complications of venous congestion resulting in compressive ischemia such as rhabdomyolysis and early compartment syndrome, a serum creatinine kinase (CK) level was ordered.

**CPC-EM Capsule**

What do we already know about this clinical entity?

Venous thoracic outlet syndrome, also known as Paget’s Schroetter’s syndrome, can present acutely when mechanical compression of stenosed veins results in a subsequent effort thrombosis.

What makes this presentation of disease reportable?

In this case, the acute presentation was obscured by the patient’s baseline sensory deficits to sensation and pain, resulting in the development of localized rhabdomyolysis.

What is the major learning point?

Ultrasound findings of interstitial edema and disorganized muscle fibers can point to a diagnosis of rhabdomyolysis or localized myositis.

How might this improve emergency medicine practice?

Performing point-of-care ultrasound or to reviewing images obtained by the technician may allow incorporation of clinical context and may lead to an expanded differential diagnosis.

Additional laboratory results revealed an elevated D-dimer 1860 milligrams per milliliter (mg/mL) (normal limit <500 mg/mL) and CK 7990 units per liter (U/L) (normal limit 30–223 U/L). With these results, the concern for complications such as rhabdomyolysis or compartment syndrome rose. The patient was started on intravenous fluids and aspirin. CT venogram revealed chronic severe luminal stricture of the left axillary vein. The rest of the veins of the chest and upper extremities were widely patent. CT angiogram was negative for PE or any arterial abnormality. The patient was admitted to the hospital with vascular surgery consultation.

During her inpatient stay, CK trended down from 7990 U/L to 1094 U/L over the next three days. The patient’s arm swelling improved throughout her hospitalization and remained well perfused without signs of worsening limb ischemia or compartment syndrome. Vascular surgery recommended no acute surgical intervention, discontinuation of use of the etonogestrel ethinyl estradiol vaginal ring, and a short course of aspirin. They arranged for close outpatient follow-up. Her case was discussed during vascular surgery case conference, a weekly educational conference in which attending and resident physicians discuss and provide recommendations for complex cases. Given her symptoms had resolved on re-evaluation two months after her admission, vascular surgery opted for continued conservative management and monitoring with repeat CT venograms.

## DISCUSSION

Initially this patient presentation prompted concern for DVT of the upper extremity, which included a limited work up with CBC, BMP, coagulation panel, ECG, and LUE DVT ultrasound. When the work-up results were negative, reevaluation with POCUS allowed the physician to visualize abnormalities. Ultrasound findings revealed interstitial edema in the soft tissues, between muscle bellies and adjacent to vasculature. In addition, disorganized muscle fibers with surrounding fluid were seen, raising the suspicion for rhabdomyolysis or, more rarely, a localized inflammatory myositis.^7^ It was suspected that a more proximal venous occlusion resulting in distal congestion was the cause, prompting additional lab tests with D-dimer and CK.

Given an elevated D-dimer at this point had resulted, the decision was made to add on both CT angiogram to assess for PE and CT venogram to assess for more proximal DVT that might have been missed on ultrasound. Furthermore, ultrasound is often technician-dependent and relies on radiological interpretation. Given upper extremity ultrasounds for DVT are less commonly performed compared to lower extremity ultrasound for DVT, there is a greater possibility of technical and interpretative error. The expanded work-up with CT venogram led to the diagnosis of an axillary vein stenosis, resulting in venous congestion of the upper extremity, and localized rhabdomyolysis.

Initial management included intravenous fluids, aspirin and surgical consultation. Had there been a definitive thrombosis identified on imaging, heparin could have been initiated. Other complications, just as with any thrombosis, may include phlegmasia alba dolens, venous gangrene, and embolization. The extent of the DVT may also indicate the need for further consultations with interventional radiology for thrombectomy vs catheter-directed thrombolysis.

Initially, venous thromboembolism was a consideration given the patient had recently started an estrogen-releasing vaginal ring lending to a hypercoagulable state. However, the cause of the patient’s venous stenosis was likely attributable to anatomic compression in the form of venous thoracic outlet syndrome. Although there was no clearly defined association, the patient’s scoliosis and history of cervical spinal fusion may have provided some rationale for the possibility of abnormal anatomy resulting in a compression or stenosis of her axillary vein. Furthermore, given her baseline sensory deficits, occult injury to the patient’s upper extremity could have led to an anatomic deformity resulting in compression of the vein and acute occlusion within the thoracic outlet.

In this case, the patient attributed her left upper extremity sensory deficits to her cervical spinal fusion. However, chronic neurogenic thoracic outlet syndrome with an acute on chronic venous thoracic outlet syndrome was also considered. Neurogenic thoracic outlet syndrome accounts for 95–99% of all cases of thoracic outlet syndrome, while venous and arterial cases account for 3–5% and 1–2%, respectively.^8^,^9^ Furthermore, neurogenic thoracic outlet syndrome most commonly presents with symptoms such as upper extremity paresthesia (98%), neck pain (88%), trapezius pain (92%), and shoulder pain (88%), all of which the patient was found to have on subsequent interview in follow-up.^10^ However, given the patient did not have any evidence of brachial plexus compression on CT, this alternative diagnosis is less likely.

## CONCLUSION

This patient likely had venous thoracic outlet syndrome, also known as Paget-Schroetter syndrome, which can present acutely when mechanical compression of stenosed veins from occult injury, repetitive movements of the arm, or stressful positioning results in stagnation of blood flow, and thus a subsequent “effort” thrombosis.^1^ Although no definitive blood clot was identified on ultrasound or CT, it is postulated that the patient’s acute symptomatology was due to a short-lived effort thrombosis of a chronically strictured axillary vein. This is evidenced by the elevated D-dimer, but limited given the lack of specificity of D-dimer for thrombosis. Furthermore, given the patient’s symptoms resolved spontaneously without surgical intervention, a short-lived thrombosis seems the most likely explanation for her acute presentation. Although the patient denied any trauma, this was difficult to ascertain given her baseline sensory deficits. She may have been prone to occult trauma or even stressful positioning such as during sleep resulting in an acute compression to a chronically stenosed vein.

This case highlights the importance of maintaining a high clinical suspicion for rhabdomyolysis or compartment syndrome in patients with baseline sensory deficits. In addition, when imaging results are unremarkable and do not correlate with the clinical picture, have a low threshold to apply POCUS to further reveal specific concerns or expand the differential diagnosis. In this patient who presented with left upper extremity swelling, POCUS elucidated findings that led to further testing and treatment of rhabdomyolysis and diagnosis of Paget-Schroetter syndrome by CT venogram.

## Figures and Tables

**Image:**
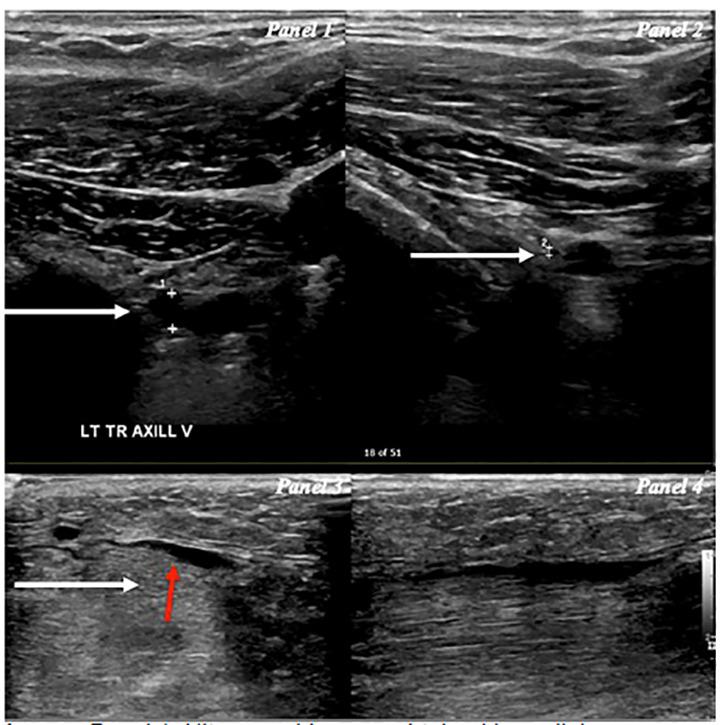
Panel 1, Ultrasound Images obtained by radiology technician: Left axillary vein in the transverse plane (white arrow), showing full compressibility (panel 2) with overlaying soft tissue swelling. Panel 3 with left upper arm with diffuse soft tissue swelling (white arrow) and interstitial edema (red arrow). Panel 4 areas of mixed echogenicity along with disorganized muscle fibers, consistent with findings of rhabdomyolysis.
